# The Intrinsic Relationship between Photoluminescence and Photocatalysis of MMoO_4_/MWO_4_ (M = Mg, Ca, Sr and Ba) Heterojunctions: Heterojunction Construction, Mechanism Insight and Development Tendency

**DOI:** 10.3390/mi15070878

**Published:** 2024-07-03

**Authors:** Man Zhang, Jagadeesha Angadi Veerabhadrappa, Shoyebmohamad Fattemohamad Shaikh, Ashok Kumar

**Affiliations:** 1 School of Electronic Engineering, Yangzhou Polytechnic College, Yangzhou 225009, China; 2 Department of Physics, P.C. Jabin Science College, Hubballi 580031, India; 3 Department of Chemistry, College of Science, King Saud University, Riyadh 11451, Saudi Arabia; sshaikh1@ksu.edu.sa; 4 Chitkara Centre for Research and Development, Chitkara University, Atal Nagar 174103, India; ashok.kumar@chitkarauniversity.edu.in

**Keywords:** photoluminescence, photocatalytic activity, MMoO_4_, MWO_4_, heterojunction

## Abstract

The migration behavior of electron and hole pairs determines both photoluminescence and photocatalytic activity, which are two distinct properties of semiconductor materials. The photoluminescence and photocatalytic activity of semiconductor materials also exhibit strong method-dependent behavior under the influence of synthesis methods. In this review, the synthesis methods of MMoO_4_, MWO_4_ and MMoO_4_/MWO_4_ (M = Mg, Ca, Sr and Ba) heterojunction composites and their photoluminescence and photocatalytic activities are reviewed for the first time. The effects of different M ions on the photoluminescence and photocatalytic activity of MMoO_4_/MWO_4_ heterojunction composites are also reviewed. There is also a discussion about the intrinsic correlation mechanism between photoluminescence and photocatalytic activity. Different M ions result in different coordination environments in MMoO_4_/MWO_4_ heterojunction composites, which leads to different photoluminescence and photocatalytic mechanisms of different MMoO_4_/MWO_4_ heterojunction composites. This review provides theoretical reference and technical guidance for future research on MMoO_4/_MWO_4_ heterojunction composites.

## 1. Introduction

The demand for lighting products is on the rise due to the rapid development of the global economy and construction industry [1]. The improvement of people’s living standards is increasingly relying on high-end lighting products to improve the quality of life [2]. At the core of high-end lighting products is the choice of luminous materials, especially luminous materials used as phosphors [3]. Until now, self-activated phosphors have received unprecedented attention for their strong luminescent properties without the need for activating ions [4]. Molybdate (MMoO_4_, M = Mg, Ca, Sr, Ba, Co, Ni, Cu et al.) and tungstate (MWO_4_, M = Mg, Ca, Sr, Ba, Co, Ni, Cu et al.) are two important self-activated phosphors, which are often used in light emitting devices, lasers, LED phosphors, Raman scattering systems, scintillators, and other fields due to their special crystal structure, electronic structure, high brightness, high fluorescence quantum yield, and high recombination rates of electron and hole pairs [5,6,7,8]. Therefore, researchers put a lot of effort into developing molybdate and tungstate luminescent devices to meet the market demand.

However, due to the low recombination rate of electron and hole pairs of single-component tungstate and molybdate, their application in the field of light-emitting devices is limited [9,10]. Recently, the following three methods have been used to enhance the luminescence efficiency of molybdate and tungstate, which has set off a new climactic point in their application in the luminescence field. (1) Surface defects of tungstate and molybdate were constructed under extreme conditions to enhance the recombination rates of electron and hole pairs [11]. (2) The optical band gap values of tungstate and molybdate were decreased by doping the A or B sites of tungstate and molybdate with the metal ions, thus enhancing the recombination rates of electron and hole pairs [12,13]. (3) Two or more semiconductor materials were combined with the tungstate or molybdate to form a heterojunction to enhance the recombination rates of electron and hole pairs [14,15,16]. The recombination rate of electron pairs for molybdate and tungstate can be effectively enhanced through heterojunction construction among these methods [17]. It is worth noting that there exists a certain correlation mechanism between the photoluminescence properties and the photocatalytic activities of tungstate- and molybdate-based luminescent materials, which promotes the research on the photocatalytic degradation of pollutants using molybdate and tungstate.

During the construction of tungstate and molybdate heterostructures, most researchers focus on the photoluminescence and photocatalytic activity of tungstate or molybdate heterostructures formed with other semiconductor materials, but few people pay attention to the physical and chemical properties of the heterostructures formed between tungstate and molybdate [18,19,20,21,22,23,24]. Therefore, some research groups focused on the photoluminescence and photocatalytic activity after the formation of a heterojunction between tungstate and molybdate. In this review, the synthesis of tungstate and molybdate and the construction of tungstate/molybdate heterojunctions are reviewed. Meanwhile, the photoluminescence and photocatalytic activity of tungstate/molybdate heterojunctions are reviewed. The influence of synthesis methods on the crystal structure, microstructure and photoluminescence properties of molybdate and tungstate has also been deeply explored. Based on these results, the internal mechanism between photocatalysis and photoluminescence is briefly reviewed and future research work is projected. From the types of photoluminescence, the internal correlation mechanism between photoluminescence and photocatalytic activity is deeply explored. These insights are helpful for subsequent studies of similar self-activated fluorescence methods as well as photoluminescent properties and photocatalytic activities of heterojunctions.

## 2. MMoO_4_/MWO_4_ (M = Mg, Ca, Sr and Ba) Heterostructure Construction

The surface morphology, particle size, dimension and physicochemical properties of tungstate and molybdate are strongly dependent on the synthesis method. Therefore, understanding the effect of synthesis methods on the physical and chemical properties of tungstate and molybdate is helpful in selecting excellent synthesis methods to synthesize tungstate- or molybdate-based phosphors with a special structure.

### 2.1. Synthesis of MMo(W)O_4_ and Ion-Doped MMo(W)O_4_

**Figure 1:**
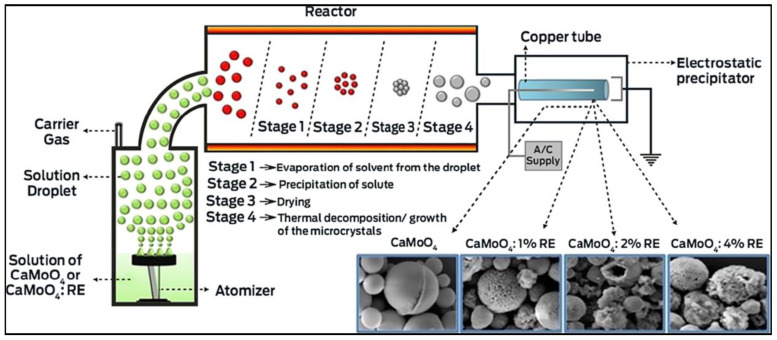
Preparation flow chart of CaMoO_4_ and CaMoO_4_:RE^3+^ phosphors using one-pot ultrasonic spray pyrolysis [38]. Adapted from ref. [38]. Copyright © 2019 American Chemical Society.

There are many methods to synthesize molybdate and tungstate, the common preparation methods including the Czochralski method, the sol–gel method, the hydrothermal method, the coprecipitation method, the self-propagating combustion method, the solid-state reaction method and so on [25,26,27,28,29,30,31,32]. When using the sol–gel method to prepare tungstate and molybdate, it is easy to obtain micron or nanoscale spherical particles and to control the phase structure and composition of the target product, which is often used for the metal ion doping of tungstate and molybdate [33]. The single crystal of tungstate and molybdate can be prepared using the Czochralski method, which can be used in lasers, LED lamps and other applications [34]. Molybdate and tungstate synthesized using the hydrothermal method are easily formed into flower shapes and other special morphologies to improve their physical and chemical properties [35]. Compared to the sol–gel method and solid phase reaction method, the temperature required for the synthesis of self-propagating combustion method is lower, and it is easier to obtain tungstate and molybdate with special defect structures. Therefore, such a method is frequently used to enhance their photoluminescence properties [36]. The solid phase reaction method is a relatively simple method that can fully mix tungsten, molybdenum and M oxides in a high temperature environment to produce MMoO_4_ and MWO_4_. However, the biggest disadvantage of this method is that the particle size is large, and the physical and chemical properties of some tungstate or molybdate at the nanometer scale are often overlooked [37]. Recently, a new method has been used to create the CaMoO_4_ and CaMoO_4_:RE^3+^ phosphors and the experimental parameters can be adjusted to control the surface morphologies and photoluminescence properties of CaMoO_4_ and CaMoO_4_:RE^3+^ phosphors, which can be regulated by adjusting the experimental parameters [38]. Figure 1 displays the preparation flow chart for CaMoO_4_ and CaMoO_4_:RE^3+^ phosphors using one-pot ultrasonic spray pyrolysis. By altering the content of RE^3+^ ions, porous spherical CaMoO_4_ and CaMoO_4_: RE^3+^ phosphors were produced.

**Figure 2:**
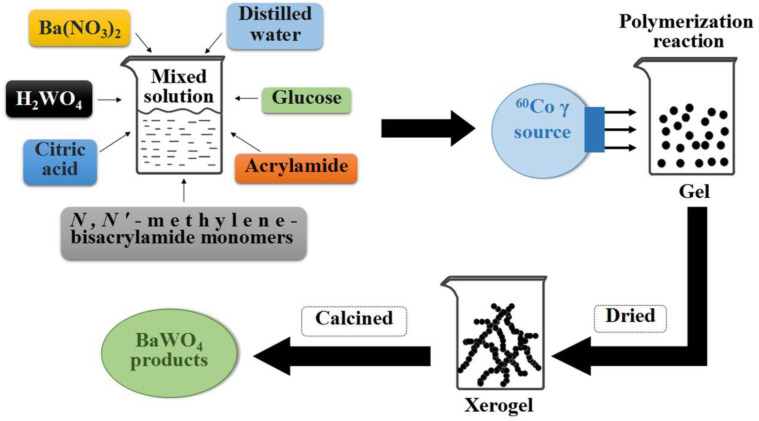
Preparation flow chart of BaWO_4_ phosphors using the polyacrylamide gel method [81]. Adapted from ref. [81]. Copyright © 2020 Wiley-VCH GmbH.

The polyacrylamide gel method is a very useful method in the synthesis of metal oxides, especially when the content of chelating agents, anti-gel collapsing agents, and crosslinking agents can be adjusted to obtain the target products with different morphologies [39]. In this method, when preparing ZnO, CeO_2_, TiO_2_ and Al_2_O_3_, the phase structure becomes more stable with the increase in sintering temperature, and the particle size increases with the increase in sintering temperature [40,41,42,43,44,45,46]. This method produces ABO_3_ oxides with superior photoluminescence or photocatalytic activity than those prepared using the conventional solid phase reaction method [47,48,49]. It is worth noting that this method has achieved key applications in the preparation of AB_2_O_4_ aluminate, chromate and ferrite, and has obtained dispersed nanoparticles with a uniform particle size [50,51,52,53,54,55,56,57,58,59,60,61]. Simultaneously, applications have also been made for the preparation of multiferroic materials with an AB_2_O_5_ structure. The nanoparticles have better magnetic properties than the bulk [62,63]. The strong magnetic hexagonal ferrites tend to agglomerate during synthesis, which reduces their magnetic properties and limits their application in magnetic memory devices. The hexagonal ferrite and hexagonal aluminate prepared using the polyacrylamide gel method have high dispersion, and some of the ferrite and aluminate appear lamellar in structure [64,65,66,67,68,69,70,71]. The ion-doped aluminate and perovskite oxides have been synthesized using the polyacrylamide gel method [72,73,74,75,76,77]. Based on the successful application of the method in the above materials, the method is also used to synthesize the MMoO_4_ and MWO_4_ [78,79,80,81,82,83,84]. Figure 2 shows the preparation flow chart of BaWO_4_ phosphors using the polyacrylamide gel method. Compared to the traditional polyacrylamide gel method, the gamma ray irradiation-assisted polyacrylamide gel method produces BaWO_4_ phosphor with a more uniform morphology than the traditional polyacrylamide gel method [81].

### 2.2. Synthesis of MMoO_4_/MWO_4_ (M = Mg, Ca, Sr and Ba) Heterojunctions

**Figure 3:**
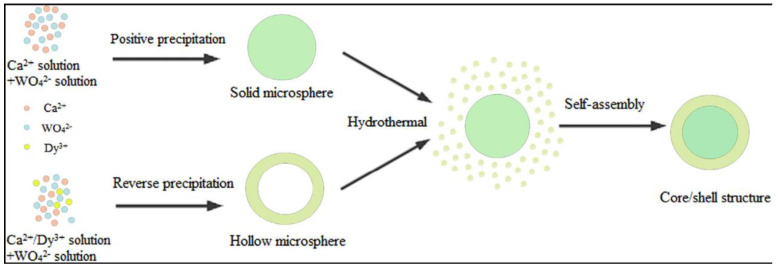
The formation process of the core/shell CaWO_4_@CaWO_4_:0.10Dy^3+^ microspheres [85]. Adapted from ref. [85]. Copyright © 2020 Wiley-VCH GmbH.

There are many methods that are used to construct a heterojunction, which can be constructed using either the synthesis of a single-component oxide or a single step synthesis method. The method of synthesizing a heterojunction of two or more metal oxides in one step using different metal sources is known as a one-step synthesis method. No matter which synthesis method is used, the initial synthesis of a single component of the metal oxide followed by another method to combine the synthesized metal oxide with another or multiple metal oxides is called a two- or multistep synthesis method [23]. Figure 3 shows the formation process of the core/shell CaWO_4_@CaWO_4_:0.10Dy^3+^ microspheres. The core/shell CaWO_4_@CaWO_4_:0.10Dy^3+^ phosphor was constructed using the positive precipitation, reverse precipitation route combined with a simple surfactant-free hydrothermal method [85]. The results show that the CaWO_4_@CaWO_4_:0.10Dy^3+^ microspheres synthesized using this method have better photoluminescence properties than those of a single component. Similarly, the core/shell CaWO_4_@CaWO_4_:Eu:Bi phosphors prepared using the low temperature reflux method also demonstrated higher photoluminescence properties than that of single-component CaWO_4_ [85].

**Figure 4:**
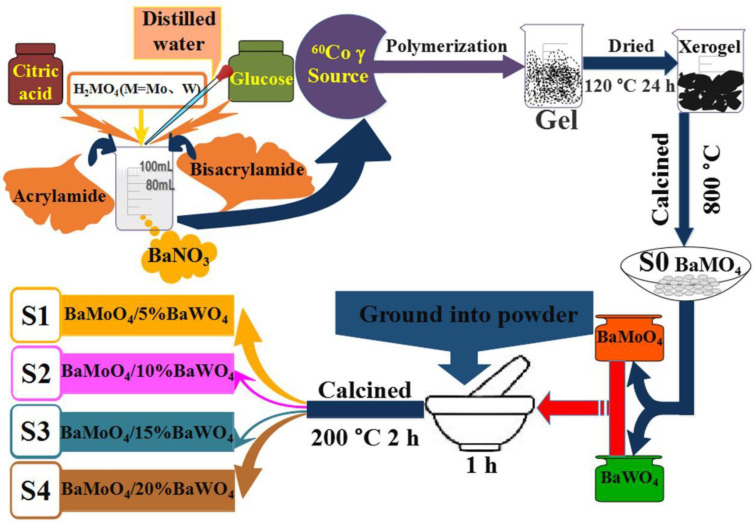
Preparation flow charts of BaMoO_4_/BaWO_4_ phosphors using the polyacrylamide gel method combined with the low temperature sintering technology [96]. Adapted from ref. [96]. Copyright © 2021 The Society of Powder Technology Japan. Published by Elsevier BV and The Society of Powder Technology Japan.

The construction of heterojunction composites can be carried out with the polyacrylamide gel method with good capacity [86,87,88,89,90,91,92,93]. The results show that the heterojunction composites synthesized using the two-step polyacrylamide gel method have higher photocatalytic activity than those synthesized using the one-step polyacrylamide gel method [94,95]. Inspired by the application of polyacrylamide gel method in the synthesis of heterojunction composites, the MMoO_4_/MWO_4_ composite phosphors were synthesized using the polyacrylamide gel method combined with the low temperature sintering technology. Figure 4 shows the preparation flow charts of BaMoO_4_/BaWO_4_ phosphors using the polyacrylamide gel method combined with the low temperature sintering technology. Firstly, MMoO_4_ and MWO_4_ were synthesized using the polyacrylamide gel method, and then they were fully ground and mixed in a specific proportion. The target products were obtained through sintering the powders above in a tubular sintering furnace at 200 °C for 2 h [96]. The advantage of this method is that the composition is easy to control, and low temperature sintering can easily form heterojunctions between different components and produce special defects, thus improving the physical and chemical properties of the system.

Table 1 shows the recently reported starting chemicals used, the method and reaction conditions of single-component MMoO_4_ and MWO_4_, and the MMoO_4_/MWO_4_ (M = Mg, Ca, Sr, Ba) heterojunction. As can be seen from Table 1, MMoO_4_ and MWO_4_ phosphors can be synthesized using the same method with different process parameters. Molybdate or tungstate phosphors with special micro-morphology such as a flower shape can be easily obtained using the hydrothermal method. The polyacrylamide gel method has become a novel method for the synthesis of metal oxide semiconductor materials, which plays an important role in the synthesis of molybdate and tungstate. The phosphors of molybdate and tungstate obtained using the polyacrylamide gel method are mainly fine nanoparticles [84]. Molybdate and tungstate prepared using the gamma ray-assisted polyacrylamide gel method are able to have special defects easily introduced to their surfaces to enhance their photoluminescence properties [79,81]. A special MMoO_4_/MWO_4_ heterojunction can be constructed by combining polyacrylamide gel method with low temperature sintering technology, which can effectively enhance the photoluminescence properties of single-component MMoO_4_ and MWO_4_ phosphors [96].

## 3. Photoluminescence Properties of MMoO_4_/MWO_4_ (M = Mg, Ca, Sr and Ba) Heterojunctions

**Figure 5:**
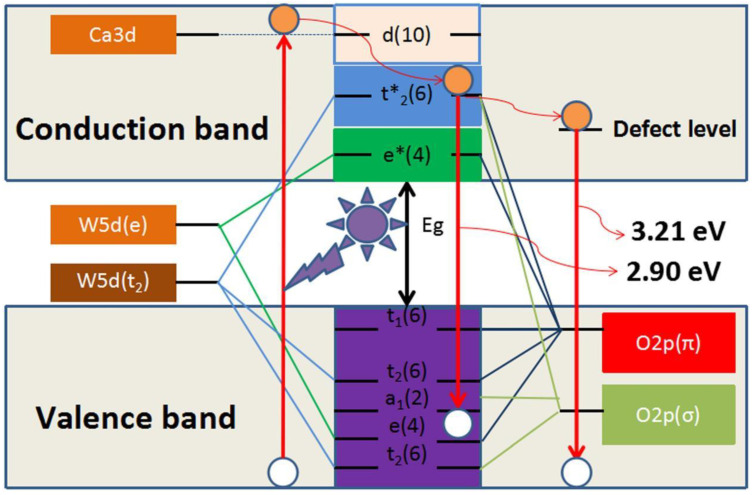
The schematic diagram of crystal-field splitting and hybridization of CaWO_4_ phosphors [79]. Adapted from ref. [79]. Copyright © 2019 Elsevier B.V.

**Figure 6:**
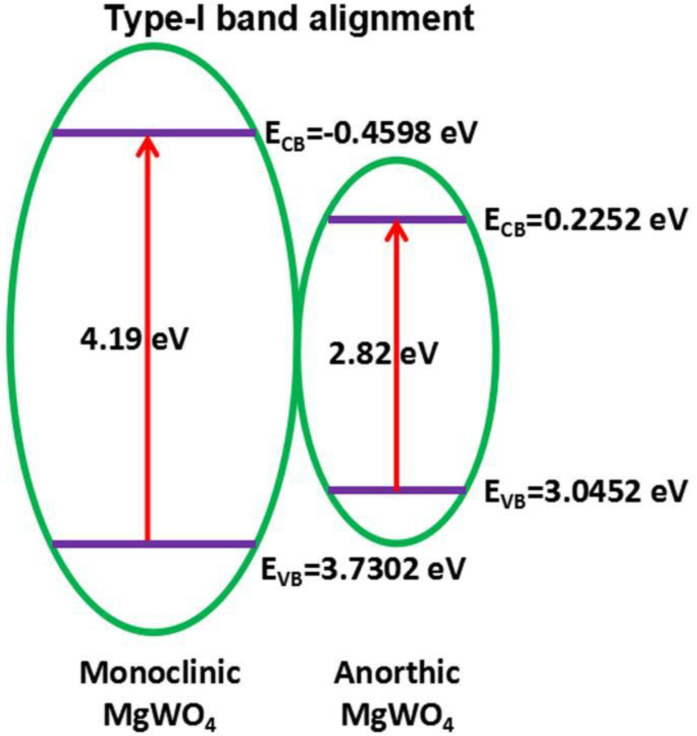
Photoluminescence mechanism of anorthic MgWO_4_/monoclinic MgWO_4_ composites [78]. Adapted from ref. [78]. Copyright © 2019 Springer Nature Switzerland AG.

Researchers have extensively studied the photoluminescence properties of MMoO_4_ and MWO_4_, which are common self-activated phosphors. A consensus has been reached on the photoluminescence mechanism of MMoO_4_ and MWO_4_ after a long period of exploration. The luminescence of single-component MMoO_4_ and MWO_4_ is mainly achieved using crystal-field splitting and hybridization. Figure 5 shows the schematic diagram of crystal-field splitting and the hybridization of CaWO_4_ phosphors. The obvious emission can be assigned to the ^1^T_2_ to ^1^A_1_ optical transition of [WO_4_]^2−^ [79,97]. The recombination rate of the charge carrier for the single-component MMoO_4_ and MWO_4_ is relatively low, which makes gives it a poor photoluminescence performance. The problem of the low recombination rate of charge carriers can be effectively solved by using a special synthesis method to construct multiple heterojunctions. Wang et al. [78] synthesized the MgWO_4_ nanoparticles using the polyacrylamide gel method, and formed anorthic MgWO_4_ and monoclinic MgWO_4_ phases in the process of high temperature sintering. A type I band arrangement is formed when the two are coupled together. Figure 6 displays the photoluminescence mechanism of anorthic MgWO_4_/monoclinic MgWO_4_ composites. The type I band arrangement structure requires that the conduction band and valence band of one kind of semiconductor are completely located in the conduction band and valence band of another kind of semiconductor. When the electrons are excited by the energy greater than the optical band gap value of MgWO_4_, the electrons and holes are easily recombined, thus greatly improving the photoluminescence performance of the system. Therefore, the construction of anorthic MgWO_4_/monoclinic MgWO_4_ heterojunction composites is advantageous in enhancing its photoluminescence properties.

**Figure 7:**
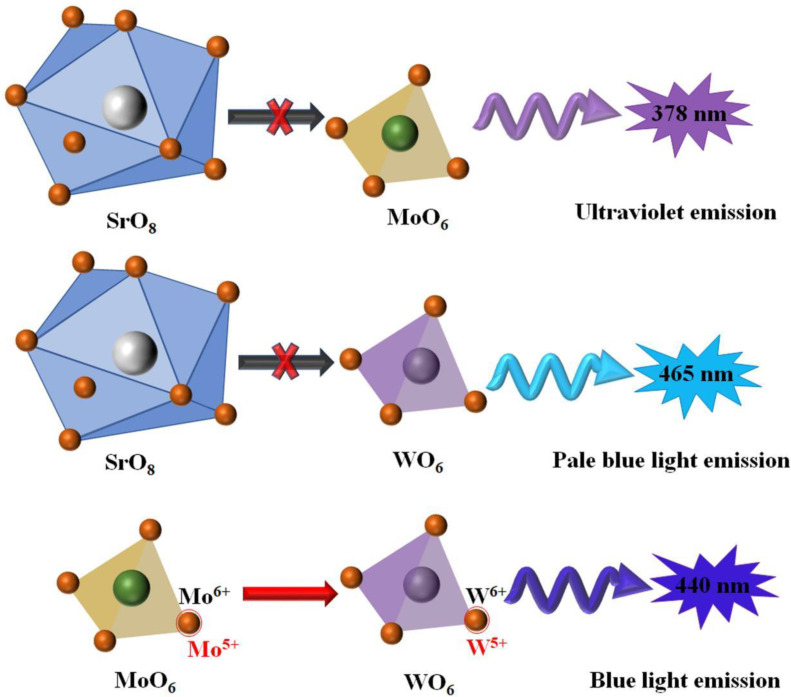
Photoluminescence mechanism of SrMoO_4_/SrWO_4_ phosphors [102]. Adapted from ref. [102]. Copyright © 2021 Elsevier B.V.

In the early years of research, Mikhailik et al. [98,99] prepared the MgWO_4_-MgMoO_4_ phosphors using the solid phase reaction method, but the Mo ion partially occupied the position of the W ion. In a strict sense, MgWO_4_ was doped by the Mo ion. It was found that the position of the fluorescence emission peak gradually moved from 500 nm to a long wavelength through the Mo ion-replacing part of the W ion. Via this excitation, our research group synthesized the MgWO_4_/MgMoO_4_ heterojunction phosphors using the polyacrylamide gel method combined with the low temperature sintering technology, and observed an obvious fluorescence emission peak at 524 nm due to the self-trapped exciton emission of the MgWO_4_ [100]. Similarly, the MMoO_4_/MWO_4_ (M = Ca, Sr, Ba) heterojunction phosphors were synthesized using the polyacrylamide gel method combined with the low temperature sintering technology and low temperature sintering did not change the phase structure of the MMoO_4_ and MWO_4_. However, the difference in the M ions resulted in different interface defects, energy level structures or impurity levels in MMoO_4_/MWO_4_ micro/nano composites, which led to the different photoluminescence and photocatalytic activities of different MMoO_4_/MWO_4_ composites, and the new luminescence properties showed a red shift phenomenon with the increase in the M ion radius. Different M metal ions with the different ionic radii and coordination number in MMoO_4_ or MWO_4_ will lead to different energy level structures of the MMoO_4_ or MWO_4_, and then lead to different photoluminescence mechanisms of the MMoO_4_/MWO_4_ (M = Mg, Ca, Sr, Ba) micro/nano heterojunction composites [96,101,102,103]. Figure 7 shows the photoluminescence mechanism of the SrMoO_4_/SrWO_4_ phosphors. According to the coordination environment of the atoms in SrMoO_4_ and SrWO_4_ and the experimental results, the charge transfer of electrons between the tetrahedrons of the MoO_4_ and WO_4_ leads to the new photoluminescence phenomenon of the SrMoO_4_/SrWO_4_ heterojunction phosphors [102].

Generally, the photoluminescence properties of a single-component MMoO_4_ or MWO_4_ and MMoO_4_/MWO_4_ (M = Ca, Sr, Ba) heterojunction mainly depend on its crystal structure, particle size, microstructure and synthesis conditions [79,96,103,104,105,106]. The existence of lattice distortion or defect in molybdate and tungstate is the main cause of their self-activated luminescence. Simultaneously, the synthesis conditions are the most influential factors to change the crystal structure, morphology and dimension of molybdate and tungstate. Extreme synthesis conditions such as ray irradiation, microwave irradiation and other methods can effectively enhance the distortion of the molybdate and tungstate lattice and enhance its photoluminescence properties [25,107,108]. Synthesis conditions such as reaction temperature, reaction time, sintering temperature, sintering time, the pH value of the precursor solution, the content of organic additives, the molar ratio of metal ions, etc., have a great impact on the size, dimension and photoluminescence properties of molybdate and tungstate [79]. Different preparation methods will also have a great impact on the above parameters of molybdate and tungstate [109,110,111]. With the increase in sintering temperature, the particle size of molybdate and tungstate and the crystallinity increases, the specific surface area decreases, and the lattice distortion and defects in molybdate and tungstate decrease, and their photoluminescence properties weaken [112,113]. It is worth noting that the photoluminescence properties of MMoO_4_ or MWO_4_ are also different with the different metal ions of M. With the increase in the radius of M ions, the fluorescence emission spectra of MMoO_4_ or MWO_4_ show a blue shift [84].

According to the above analysis, the luminescence of a single-component MMoO_4_ or MWO_4_ and MMoO_4_/MWO_4_ (M = Ca, Sr, Ba) heterojunction is obviously different. The photoluminescence of a single-component MMoO_4_ or MWO_4_ is mainly caused by the distortion or defect of the lattice of [MoO_4_]^2-^ or [WO_4_]^2-^, which is related to the internal structure of the phosphor. The distortion of the crystal structure causes the luminescence of molybdate or tungstate, which is the reason why they can self-activate luminescence. However, the photoluminescence of the MMoO_4_/MWO_4_ (M = Ca, Sr, Ba) heterojunction, in addition to the photoluminescence of single-component phosphors, also presents new luminescence phenomena caused by interface defects caused by interface coupling between the two phosphors, which is significantly different from the luminescence of single-component tungstate or molybdate. In other words, the luminescence phenomenon of a MMoO_4_/MWO_4_ (M = Ca, Sr, Ba) heterojunction also depends on the charge transfer and recombination rate between the interface, regarding whether to form a type I band arrangement structure.

## 4. Photocatalytic Activity of MMoO_4_/MWO_4_ (M = Mg, Ca, Sr and Ba) Heterojunctions

**Figure 8:**
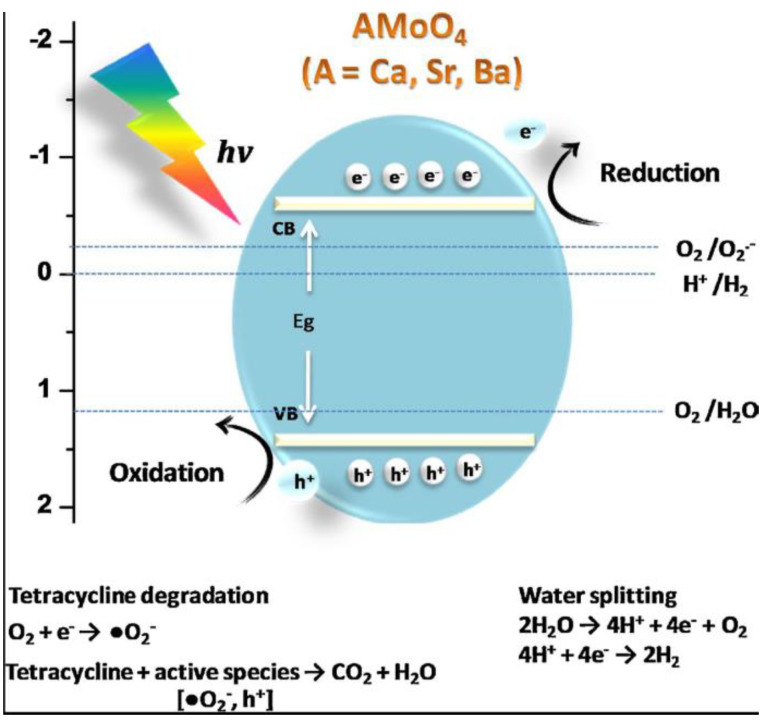
Photocatalytic mechanism of AMoO_4_ (M = Ca, Sr, Ba) materials for the degradation of tetracycline hydrochloride [120]. Adapted from ref. [120]. Copyright © 2017 Elsevier B.V.

In addition to high photoluminescence properties, MMoO_4_ and MWO_4_ also possess high photocatalytic activity. However, due to the high recombination rate of electron–hole pairs in MMoO_4_ and MWO_4_, their photocatalytic activity is low, so the relevant results are relatively few [114,115,116,117,118,119,120,121,122]. In addition to the above reasons, one of the main reasons is that the MgMoO_4_ is easily hydrolyzed, which limits its application in the field of photocatalysis. Another reason worth noting is that the MMoO_4_ and MWO_4_ only respond to UV light due to their large optical bandgap values, which limits their application in the field of photocatalysis to some extent. Therefore, solving the hydrolysis problem of the MgMoO_4_ and reducing its band gap value become the key to whether MgMoO_4_ can be used in the field of photocatalysis. Another way to inhibit the hydrolysis of MgMoO_4_ is to use it to degrade insoluble contaminants such as methyl red [82]. Figure 8 shows the photocatalytic mechanism of MMoO_4_ (M = Ca, Sr, Ba) materials for the degradation of tetracycline hydrochloride. The results show that the superoxide radicals and holes play an important role in the whole photocatalytic process [120]. It is worth noting that there is no linear dependence between the photocatalytic activity of the MMoO_4_ photocatalyst and the ionic radius of the M ion. With the increase in the ionic radius of the M ions, the crystal structure, electronic structure and energy band structure of the MMoO_4_ are different, which will greatly affect the photocatalytic activity of the MMoO_4_, in turn promoting the understanding of the details of the photocatalytic mechanism of the MMoO_4_.

**Figure 9:**
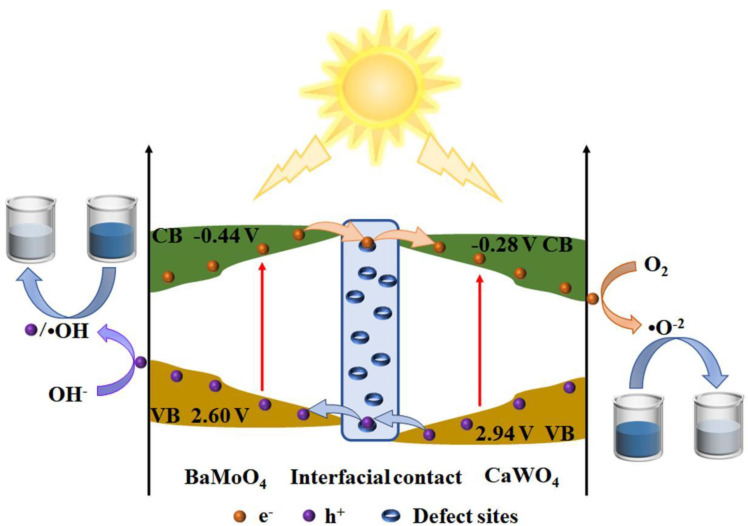
Photocatalytic mechanism of BaMoO_4_-coupled CaWO_4_ heterojunction micro/nanocomposites for the degradation of methylene blue [114]. Adapted from ref. [114]. Copyright © The Minerals, Metals & Materials Society 2022.

Due to the above shortcomings of the MMoO_4_ and MWO_4_, researchers continue to take measures to enhance their photocatalytic activity. Among them, the most important method is to couple MMoO_4_ or MWO_4_ with other semiconductor materials to form the heterojunctions to improve the transfer and separation efficiency of charge carriers in the heterojunction system, so as to enhance its photocatalytic activity for the degradation of tetracycline hydrochloride, sulfamethoxazole, organic dye and photocatalytic hydrogen production from reducing water [123,124,125,126,127,128]. The direct coupling of MMoO_4_ and MWO_4_ can also enhance their photocatalytic activity. Figure 9 shows the photocatalytic mechanism of BaMoO_4_-coupled CaWO_4_ heterojunction micro/nanocomposites for the degradation of methylene blue. When BaMoO_4_ is coupled with the CaWO_4_, a special interface contact is formed at the interface to enhance the transfer and separation efficiency of charge carriers [114]. The associated chemical processes can be described as follows [114]:

(1) Charge carrier generation:

MMoO_4_/MWO_4_ + h → e_CB_^−^ + h_VB_^+^ (1)

(2) Hydroxyl free radical and superoxide free radical production in valence band:

2e_CB_^−^ + O_2_ + 2H^+^ → H_2_O_2_ (2)

e_CB_^−^ + H_2_O_2_ → •OH (Hydroxyl free radical) + OH^−^ (3)

e_CB_^−^+O_2_→•O_2_^−^ (Superoxide free radical) (4)

•O_2_^−^+ 2H^+^ +e_CB_^−^→H_2_O_2_ (5)

•O_2_^−^+H_2_O_2_→•OH + OH^−^+O_2_ (6)

(3) Hydroxyl free radical production in conduction band:

h_VB_^+^ + H_2_O → •OH + H^+^ (7)

h_VB_^+^ + OH^−^ → •OH (8)

(4) Pollutant degradation:

•OH + Pollutant → degradation products(9)

h_VB_^+^ + Pollutant → degradation products (10)

Similarly, a similar phenomenon has been observed in CaMoO_4_/CaWO_4_ heterojunction micro/nanocomposites for the degradation of methylene blue [101]. However, the opposite phenomenon was found in SrMoO_4_/SrWO_4_ and BaMoO_4_/BaWO_4_ heterojunction micro/nanocomposites for the degradation of methylene blue [96,102]. Different M ions lead to different photocatalytic activities of the MMoO_4_/MWO_4_ heterojunction composites, which will make the photocatalytic mechanism of this series of composites more complicated and require more studies to explain its photocatalytic mechanism in the future.

Single-component MMoO_4_, MWO_4_ and MMoO_4_/MWO_4_ (M = Ca, Sr, Ba) heterojunctions are mainly used to degrade organic dyes, pharmaceuticals and other pollutants that are difficult to degrade naturally. Affected by environmental factors such as catalyst content, contaminant concentration and pH value of reaction solution, the best degradation parameters exist when the photocatalyst degrades the contaminant. In the past, the method to find the optimal experimental parameters is to perform a lot of experiments through trial and error, which is not only time-consuming but is also not necessarily able to find the appropriate parameters. With the development of artificial intelligence technology, researchers have adopted intelligent algorithms to find the best experimental parameters, which can not only obtain the best degradation parameters, but also develop new photocatalysts [129,130,131].

## 5. The Intrinsic Correlation Mechanism between Photoluminescence and Photocatalytic Activity

**Figure 10:**
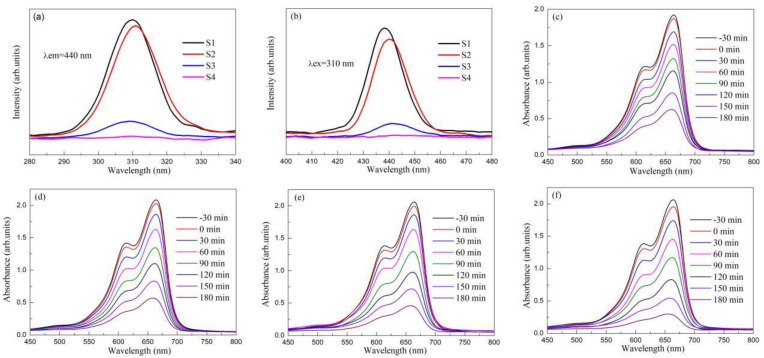
(**a**) Excitation spectra and (**b**) emission spectra of SrMoO_4_/SrWO_4_ micro/nano heterojunction phosphors. UV-Vis absorption spectra of (**c**) SrMoO_4_/5 wt% SrWO_4_ (S1), (**d**) SrMoO_4_/10 wt% SrWO_4_ (S2), (**e**) SrMoO_4_/15 wt% SrWO_4_ (S3), and (**f**) SrMoO_4_/20 wt% SrWO_4_ (S4) micro/nano heterojunction phosphors for the degradation of methylene blue [102]. Adapted from ref. [102]. Copyright ©2021 Elsevier B.V.

The relationship between the emission intensity and photocatalytic activity of semiconductor materials is very complicated, mainly depending on whether the semiconductor material is doped, whether the heterojunction is constructed and whether the surface defects are produced. When the semiconductor material is doped with a certain amount of impurity ions, the content of impurity ions has a great effect on the photoluminescence and photocatalytic activity, mainly affecting the transfer and separation efficiency of the charge carriers. The stronger the fluorescence signal of the semiconductor material, the higher the content of oxygen vacancies and defects on its surface, and the higher the photocatalytic activity. When MMoO_4_ and MWO_4_ are coupled together, there is an optimal ratio between them that makes it easy recombine or separate electron and hole pairs, which determines whether there is an energy barrier between the MMoO_4_/MWO_4_ heterojunction, preventing the recombination between electron and hole pairs and thus improving the photocatalytic activity of the system [96,101,114]. The photocatalytic activity in the MMoO_4_/MWO_4_ heterojunction is weaker when it has higher emission intensity [96,101,114]. Therefore, the detailed measurements of the photoluminescence properties of MMoO_4_/MWO_4_ heterojunctions can provide an effective insight into their photocatalytic activities, which will help the advancement of photocatalytic technology. Figure 10 shows the dependence of photocatalytic activity on the photoluminescence spectra of SrMoO_4_/SrWO_4_ micro/nano heterojunction phosphors. As can be seen from Figure 10, there is a linear dependence between emission intensity and photocatalytic activity [102]. In other words, the photocatalytic activity of MMoO_4_/MWO_4_ heterojunction phosphors decreases with an increase in emission intensity. In spite of the above conclusions obtained from the literature, the mechanism of the MMoO_4_/MWO_4_ heterojunction phosphors is also different due to the different effects of the M ions on their photoluminescence and photocatalytic activities, which will make it very difficult to explore the internal mechanism of their photoluminescence and photocatalytic activities. In future work, it is necessary to conduct many experiments to verify the linear relationship between the photoluminescence and photocatalytic activity of MMoO_4_/MWO_4_ heterojunction phosphors.

It is worth noting that photoluminescence is divided into discrete luminescence and recombination luminescence [132]. The discrete luminescence is mainly related to the internal structure of the semiconductor phosphor, while the recombination luminescence is mainly related to the recombination rate of the electron–hole pairs of the semiconductor phosphor [133]. The discrete luminescence is mainly caused by excitons, and the recombination of charge carriers does not occur [134]. Recombination luminescence mainly requires the rapid recombination of electron–hole pairs and the excess energy can be emitted in the form of photons. The photocatalytic process is the process of using light energy to stimulate the separation of electrons and hole pairs. The photocatalytic activity of semiconductor materials will be enhanced only with the increase in electron and hole transfer and separation efficiency. If the photocatalyst can also produce a luminescence phenomenon, and the luminescence phenomenon is caused by discrete luminescence, then the photocatalytic activity of the semiconductor material is proportional to the photoluminescence properties [135,136]. If the luminescence of the photocatalyst is caused by recombination luminescence, the photocatalytic activity becomes worse with the increase in emission peak intensity [96,101,103,137,138,139]. Therefore, to explore the internal correlation mechanism between photocatalytic activity and photoluminescence properties of photocatalysts, it is necessary to deeply understand the mechanism of photoluminescence in photocatalysts.

**Figure 11:**
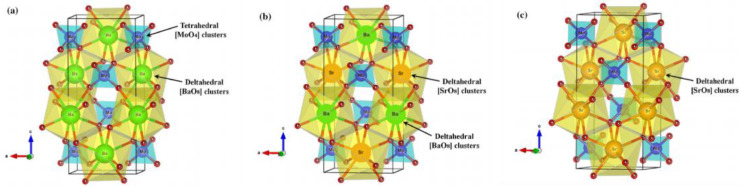
Effects of different coordination numbers on the structure of (**a**) BaMoO_4_, (**b**) (Ba_0.5_Sr_0.5_)MoO_4_ and (**c**) SrMoO_4_ unit cells [140]. Adapted from ref. [140]. Copyright ©Springer Science+Business Media New York 2015.

The difference in M ions means that they have different ionic radii and different coordination numbers from MoO_4_ and WO_4_. The lattice distortion of MMoO_4_ and MWO_4_ varies with different M ions, which results in significant changes in their photoluminescence and photocatalytic activity. Figure 11 displays the effects of different coordination numbers on the structure of BaMoO_4_, (Ba_0.5_Sr_0.5_)MoO_4_ and SrMoO_4_ unit cells [140]. Different coordination environments bring about different lattice distortions in the unit cells, and show different performance characteristics in terms of photoluminescence and photocatalytic activity [141]. When MMoO_4_ and MWO_4_ are coupled together to form a heterojunction, in addition to the fluorescence emission peak caused by lattice distortion, the interface defect makes them produce a new fluorescence emission peak [102,103]. However, in order to clearly analyze the mechanism of the internal correlation between the photoluminescence and photocatalytic activity of the MMoO_4_/MWO_4_ heterojunction, further details are needed, especially as the charge carrier recombination rate between the interface is unclear. In future research work, the construction of special MMoO_4_/MWO_4_ heterojunctions through first-principles calculation and the study of their photoelectric properties will provide theoretical support for this series of heterojunctions.

## 6. Conclusions and Prospects

The internal correlation mechanism between the photoluminescence and photocatalytic activity of MMoO_4_/MWO_4_ heterojunction micro/nano composites has been the focus of considerable research. The synthesis methods of the MMoO_4_, MWO_4_ and MMoO_4_/MWO_4_ heterojunction composites are reviewed. These results confirm that the photoluminescence and photocatalytic activity of the MMoO_4_, MWO_4_ and MMoO_4_/MWO_4_ heterojunction composites are strongly dependent on the synthesis methods. Meanwhile, the photocatalytic activity and photoluminescence properties of the MMoO_4_/MWO_4_ heterojunction composites with the different M ions are reviewed. The results show that there is no linear relationship between the photocatalytic activity and photoluminescence of MMoO_4_/MWO_4_ heterojunction composites and M ions. The luminescence intensity of MMoO_4_/MWO_4_ heterojunction composites was reduced as photocatalytic activity increased.

The development trends for MMoO_4_/MWO_4_ heterojunction composites will be as follows: (1) Enhancing the transfer and separation efficiency of electron and hole pairs in MMoO_4_/MWO_4_ heterojunction composites can be achieved by introducing electron and hole carriers between MMoO_4_ and MWO_4_, which improves the photocatalytic activity of the system. (2) An intelligent algorithm optimized neural network model was introduced to train the experimental results of the photoluminescence and photocatalytic activities of existing MMoO_4_/MWO_4_ heterojunction composites, and then predict their photoluminescence and photocatalytic activities, providing theoretical guidance for the development of new efficient phosphors or photocatalysts. (3) Special unit cells were constructed, and the electronic structure, band structure and electronic state density of MMoO_4_/MWO_4_ heterojunction composites were calculated and simulated using first-principles. The results were compared with the experimental results and guided the experiment. (4) The photoluminescence and photocatalytic activities of MMoO_4_/MWO_4_ heterojunction composites are significantly different with different M ions. The combination of three or more molybdate and tungstate salts can be investigated for their photoluminescence and photocatalytic activity. (5) The photoluminescence or photocatalytic activity of MMoO_4_/MWO_4_ heterojunction composites can be enhanced by constructing special heterojunctions with special defect structures using certain extreme conditions. (6) Ion doping MMO_4_ or MWO_4_ coupled with the MMO_4_ or MWO_4_ to enhance the photoluminescence or photocatalytic activity of the system is also a hot research direction in the future.

## Figures and Tables

**Table 1:** The recently reported starting chemicals used, method and reaction conditions of single-component MMoO_4_ and MWO_4_, and MMoO_4_/MWO_4_ (M = Mg, Ca, Sr, Ba) heterojunction.

Sample	Synthesis Method	Starting Chemicals	Reaction Conditions	Refs.
CaMoO_4_	The solid-state diffusion method	Calcium carbonate (CaCO_3_), ammonium molybdate([NH_4_]_6_Mo_7_O_24_·4H_2_O) and polyethylene glycol [PEG] HO(CH_2_-CH_2_O)_n_H	Sintered at 900 °C for 1.5 h	[25]
CaMoO_4_:Eu	The sol–gel method	CaCl_2_ (96%), (NH_4_)_6_Mo_7_O_24_·4H_2_O (99%), CaCO_3_ (99%), MoO_3_ (99%) and Eu_2_O_3_ (99.99%)	Sintered at 800 °C for 3 h	[26]
CaMoO_4_	Chemical precipitation method	Ca(NO_3_)_3_, Na_2_MoO_4_	Sintered at 800 °C for 1 h	[27]
CaMoO_4_	Hydrothermal method	Ethylene glycol, Ca(NO_3_)_2_·4H_2_O, Na_2_MoO_4_·2H_2_O, HNO_3_	160 °C for 16 h	[30]
BaMoO_4_	Hydrothermal method	Ba(NO_3_)_2_, Na_2_MoO_4_·2H_2_O, HNO_3_	220 °C for 24 h	[35]
BaMoO_4_	Auto-igniting combustion technique	Ba(NO_3_)_2_, MoO_3_, HNO_3_	Sintered at 750 °C for 3 h	[36]
CaWO_4_	Polyacrylamide gel method	Ca(NO_3_)_2_·4H_2_O (99%), H_2_WO_4_, citric acid, glucose, acrylamide and N, N’-methylene-bisacrylamide	Sintered at 800 °C for 5 h	[79]
BaWO_4_	Polyacrylamide gel method	Ba(NO_3_)_2_, H_2_WO_4_, citric acid, glucose, acrylamide and N, N’-methylene-bisacrylamide	Sintered at 800 °C for 5 h	[81]
MMoO_4_ (M = Mg, Ca, Sr)	Polyacrylamide gel method	Mg_2_(OH)_2_CO_3_, CaCO_3_, SrCO_3_, H_2_MoO_4_, citric acid, glucose, acrylamide and N, N’-methylene- bisacrylamide	Sintered at 800 °C for 5 h	[84]
CaWO_4_@ CaWO_4_: Dy^3+^	A simple surfactant-free hydrothermal route	Ca(NO_3_)_2_·4H_2_O, Na_2_WO_4_·2H_2_O and Dy(NO_3_)_3_·4H_2_O	120 °C for 12 h	[85]
BaMoO_4_/BaWO_4_	Polyacrylamide gel method combined with low temperature sintering technology	BaMoO_4_ and BaWO_4_	200 °C for 2 h	[96]

## Data Availability

The original contributions presented in the study are included in the article, further inquiries can be directed to the corresponding author.
